# Inactivation of DNA repair—prospects for boosting cancer immune surveillance

**DOI:** 10.1186/s13073-018-0603-9

**Published:** 2018-11-28

**Authors:** Anna Truini, Giovanni Germano, Alberto Bardelli

**Affiliations:** 1 Candiolo Cancer Institute, FPO-IRCCS, Candiolo, TO Italy; 2 Department of Oncology, University of Torin, SP 142 km 3.95, 10060 Candiolo, TO Italy

**Keywords:** Tumor evolution, Mismatch repair, MMR, Tumor, Mutational burden, Neoantigen, Immune surveillance

## Abstract

The emergence of drug resistance depends on the ability of the genome of cancer cells to constantly mutate and evolve under selective pressures. The generation of new mutations is accelerated when genes involved in DNA repair pathways are altered. Notably, although the emergence of new mutations fosters drug resistance, new variants can nevertheless become novel antigens that promote immune surveillance and even restrict cancer growth.

## Cancer evolution and tumor mutational burden

The ability of tumors to adapt to drug pressure depends on the capacity of cancer cells to evolve over time. These observations raise the important issue of how treatment plans can overcome the near-certainty of disease relapse. Until recently, much of the effort has been directed at preventing or restricting tumor evolution. However, understanding the ways tumors mutate and how this affects immune surveillance and immune responses could be a more effective approach for cancer therapies.

Tumors evolve owing to their inherent genetic instability and molecular heterogeneity. Cancers encompass different cellular populations carrying distinct genetic and epigenetic alterations and are capable of continuously acquiring new somatic variants. Several lines of evidence indicate that tumors deficient in mismatch repair (MMR)—characterized by hypermutability and an increased mutation rate—are highly responsive to immune-checkpoint inhibitors [1, 2]. This observation led to the hypothesis that an increased mutation load (number of mutations per megabase) could increase the efficacy of immunotherapy. Indeed, malignancies whose etiologies correlate with environmental exposure, such as melanoma and lung cancers, which are characterized by a high tumor mutational burden (TMB), have been shown to be particularly sensitive to immunotherapy [3].

Rizvi and colleagues have reported how a higher non-synonymous mutation burden is associated with improved response, durable clinical benefit, and progression-free survival in two independent cohorts of non-small-cell lung cancer patients [1]. In their study, the efficacy of the treatment also correlated positively with a higher neoantigen burden. Particular attention has also been paid to the expression of programmed death-ligand 1 (PD-L1) in the tumor microenvironment, which is thought to reflect the activity of effector T cells. In a large clinical data-set, it was even clearer that the expression of PD-L1 in pre-treatment biopsies identified patients most likely to benefit from inhibitors of programmed cell death protein 1 (PD-1) and/or its ligand PD-L1 [4]. PD-L1 expression also strongly correlates with various markers of active cellular immunity responders [5]. In patients with both high TMB and PD-L1 positivity, a durable response rate of 50% was observed, suggesting that combining these variables can improve the ability to predict responses to checkpoint inhibitors [6]. Anti-PD-1-induced neoantigen-specific T-cell reactivity can also be detected in the blood, and this might lead to the development of blood-based tests to monitor responses during administration of immune-checkpoint inhibitors [1].

## Inactivation of MMR and the response to immune checkpoint blockade

How MMR deficiency affects the response to immunotherapy has been unraveled in recent work demonstrating that tumors containing a high number of somatic mutations, owing to defects in DNA MMR, are sensitive to immune-checkpoint blockade—with anti-PD-1 antibodies—across 12 different tumor types [2]. Objective responses were reported in more than half of the patients, with 21% being complete responses. Of note, this study also demonstrated a rapid in vivo expansion of neoantigen-specific T-cell clones reactive to tumor mutant neopeptides. This finding strongly supports the idea that mutant neoantigens are responsible for sensitivity to immunotherapy [2].

Overall these studies underline the concept that a high mutational burden and elevated number of neoantigens, due to alterations in MMR genes, renders tumors responsive to immunotherapy regardless of the type of cancer. Based on these lines of evidence, the FDA granted approval to the anti-PD-1 antibody pembrolizumab in advanced solid tumors in patients whose cancer is DNA-MMR deficient [7]. This is the first example of approval of a ‘tissue-agnostic’ treatment based on the cancer biomarker status, rather than on tumor histology.

Although immunotherapy has shown promising results, unfortunately it is effective in only a minority of cancer patients, and thus there is an intense interest towards understanding why immunotherapeutic approaches might differentially benefit distinct patient subgroups.

## Immune-cold and immune-hot tumors

A major limitation of the efficacy of immunotherapy is represented by the so-called ‘immunologically cold tumors’. This term usually refers to there being limited, or no, immune response within the tumor tissue. Cold tumors are those that are not recognized by the innate or adaptive immune system, and do not elicit strong immune responses. Different cold immune profiles have been identified by analysis of histological samples. The ‘immune-excluded’ phenotype is characterized by the presence of abundant immune cells that, however, do not infiltrate the parenchyma but instead remain in the stroma of the tumor mass [5]. A second profile, the ‘immune-desert’ phenotype, is characterized by the absence of T cells in both the parenchyma and the stroma of the tumor. A third group is represented by inflamed tumors that contain a large repertoire of CD8^+^, CD4^+^, and myeloid cells as well as a complex network of pro-inflammatory cytokines [5]. Such strong intra-tumoral immune abundance (that suggests a pre-existing immune response) is blocked by the inhibitory strategies of the tumor, preventing effective immune surveillance. In addition, low levels of neoantigens, as well as the secretion of immunosuppressive cytokines, are also typical features of immunologically cold tumors.

Several strategies have been considered to increase immune surveillance of cold tumors. For example, the impact of MMR deficiency on cancer immune surveillance was assessed recently by using syngeneic mouse models [8]. Specifically, the gene encoding MutL homologue 1 (MLH1), a key component of the DNA MMR system, has been genetically inactivated in colorectal, breast, and pancreatic mouse cancer cells. While the growth of MMR-deficient cancer cells in immunocompromised mice was comparable to that of their proficient counterparts, MMR-deficient cells grew poorly when transplanted into immunocompetent mice [8]. Indeed, inactivation of MMR not only significantly increased the mutational burden but also led to a persistent renewal of neoantigens compared with MMR-proficient cells. This resulted in improved immune surveillance and restricted tumor growth.

Combined, these results suggest that a forced increase in the mutational burden (particularly the levels of frameshifts) could, paradoxically, be beneficial. As a follow-up, colorectal cancer mouse cell lines were treated with temozolomide (TMZ), a chemotherapeutic drug that triggers DNA damage, and TMZ-resistant cells were injected into syngeneic mice [8]. A subset of drug-resistant cells (those that had lost MMR capabilities) did not form tumors and showed an increased number of mutations and number of predicted neoantigens in comparison with parental cell lines. Taken together, these results showed that an increased mutational load, triggered by inactivation of MMR and associated with hypermutability, can initiate an effective immune response.

Another approach to increase the response of cancers to immunotherapies is delivery of oncolytic viruses. For example, a small phase Ib clinical trial recently tested the effect of combining an oncolytic virus with the anti-PD-1 pembrolizumab in a cohort of advanced melanoma patients. This combination reported an impressive 62% overall response rate, with 33% being complete responses [9]. The strategy led to reprogramming of the microenvironment and T-cell infiltration in tumors, which effectively turned an immunologically cold tumor into an immune hot tumor [9].

A further option is represented by a personalized cancer treatment vaccine that targets patient-specific neoantigens. Ott and colleagues recently showed how this approach could stimulate an effective anti-tumor response in melanoma patients. When the vaccine was injected into the patients, it drove immune T-cell responses that recognized neoantigens on tumor cells, resulting in complete responses [10]. Oncogenic driver mutations are required for the tumor growth, but they can also be considered as tumor-specific neoantigens, shared among patients. Rosenberg and colleagues evaluated the reactivity of tumor-infiltrating CD8^+^ T lymphocytes that specifically recognized KRAS harboring the G12D mutation in a patient with multiple metastatic sites. After the lymphocytes were expanded and infused, an objective regression was observed for all seven lung metastases [11].

Other strategies to convert an immune-cold tumor into an immune-hot target include the use of nanoparticles capable of delivering immune-stimulating drugs into tumors and, in turn, stimulating T-cell invasion. Reprogramming the tumor microenvironment to elicit T-cell activation and enhance tumor immunity represents another way to effectively convert cold tumors into hot tumors. Finally, small-molecule drugs that increase T-cell activity could act synergistically with immune-checkpoint inhibitors.

## Concluding remarks

Up until now, significant efforts have been made in the direction of preventing and/or restricting tumor evolution for therapeutic strategies. An alternative approach is to increase the immunogenicity of cancer cells, thus fostering immune surveillance. In this regard, several strategies have been proposed, including, first, inactivation of DNA repair to increase neoantigen levels in cancer cells; second, modification of the tumor microenvironment; and finally delivering tumor-specific viruses. These approaches are being tested in preclinical models or in early-stage clinical experimentation, with the ultimate goal of improving immune surveillance and restricting cancer growth.

## Abbreviations

Anti-PD-1: Anti-programmed cell death protein 1
MMR: Mismatch repair
PD-L1: Programmed cell death 1 ligand 1
